# Two-eyed seeing of the integration of oral health in primary health care in Indigenous populations: a scoping review

**DOI:** 10.1186/s12939-020-01195-3

**Published:** 2020-06-30

**Authors:** Richa Shrivastava, Yves Couturier, Felix Girard, Lucie Papineau, Elham Emami

**Affiliations:** 1 Faculty of Dentistry, Université de Montréal, Montréal, Québec H3C 3J7 Canada; 2 School of Social Work, Université de Sherbrooke, Sherbrooke, J1H 4C4 Québec Canada; 3 Cree Board of Health and Social Services of James Bay, Oujé-Bougoumou Healing Centre, Oujé-Bougoumou, Québec G0W 3C0 Canada; 4 Faculty of Dentistry, McGill University, Montreal, Québec H3A 1G1 Canada

**Keywords:** Primary health care, Dental care, Integrated health care systems, Indigenous populations, Two-eyed seeing

## Abstract

**Background:**

Indigenous people experience significant poor oral health outcomes and poorer access to oral health care in comparison to the general population. The integration of oral health care with primary health care has been highlighted to be effective in addressing these oral health disparities. Scoping studies are an increasingly popular approach to reviewing health research evidence*.* Two-eyed seeing is an approach for both Western and Indigenous knowledge to come together to aid understanding and solve problems. Thus, the two-eyed seeing theoretical framework advocates viewing the world with one eye focused on Indigenous knowledge and the other eye on Western knowledge. This scoping review was conducted to systematically map the available integrated primary oral health care programs and their outcomes in these communities using the two-eyed seeing concept.

**Methods:**

This scoping review followed Arksey and O’Malley’s five-stage framework and its methodological advancement by Levac et al. A literature search with defined eligibility criteria was performed via several electronic databases, non-indexed Indigenous journals, Indigenous health organizational websites, and grey literature. The charted data was classified, analyzed, and reported using numeral summary and qualitative content analysis. The two-eyed seeing concept guided the interpretation and synthesis of the evidence on approaches and outcomes.

**Results:**

A total of 29 publications describing 30 programs conducted in Australia and North America from 1972 to 2019 were included in the final analysis. The following four program categories emerged from the analysis: oral health promotion and prevention programs (*n* = 13), comprehensive dental services (n = 13), fly in, fly out dental services (*n* = 3), and teledentistry (n = 1). Biomedical approaches for integrated primary oral health care were leadership and governance, administration and funding, capacity building, infrastructure and technology, team work, and evidence-based practice. Indigenous approaches included the vision for holistic health, culturally appropriate services, community engagement, shared responsibility, and cultural safety. The program outcomes were identified for biological, mental, and emotional dimensions of oral health; however, measurement of the spiritual dimension was missing.

**Conclusion:**

Our results suggest that a multiple integrated primary oral health care approach with a particular focus on Indigenous culture seems to be efficient and relevant in improving Indigenous oral health.

## Background

Indigenous people account for approximately 6% of the overall world population [1]. Around the world, these populations experience significant poor oral health outcomes and poorer access to oral health care in comparison to general populations [2–6]. Indigenous people face barriers related to the impact of colonization and government assimilation policies, discrimination and subsequent marginalization, lifestyle and dietary modifications, lack of understanding of their cultural values, and provision of culturally inappropriate services [7–11]. Those living in rural and remote areas further encounter barriers to oral health care such as access to and availability of dental services, shortage and accessibility of dental professionals, geographical remoteness, poor socioeconomic status, travel difficulties, infrastructure deficit, and diminished dental insurance coverage [12–14]. Furthermore, the fragmentation of health care and the disconnection between dental and medical care have aggravated the undue burden of oral disease and poor access to care in Indigenous people [15].

These disparities also confirm the failure of conventional health services in adequately serving Indigenous health care needs [16]. Hence, the integration of oral health care with primary health care has been highlighted to be effective in addressing oral health disparities among Indigenous communities [17–20]. Integrated care is emphasized as one of the basic concepts of primary care, and defined as *a coherent and coordinated set of services which are planned, managed, and delivered to individual service users across a range of organizations and by a range of co-operating professionals and informal carers* [21]. Integration of oral health into primary health care is more acceptable for Indigenous populations as it has the potential to incorporate Indigenous values and principles, and management by Indigenous people, in addition to comprehensive service delivery [16, 22, 23]. As per the Aboriginal Mental Health best practices working group, integration is a concept that *completes the circle of care* [24, 25].

Accordingly, some primary health care organizations serving Indigenous populations, such as Indian Health Services in the United States, First Nations and Indian Health Services in Canada, and Aboriginal Community Controlled Health Services in Australia, have integrated oral health care into their services [16]. Several action plans and strategies have been developed in these countries with objectives and recommendations on integrating culturally sensitive oral health into primary care for Indigenous populations; for instance, the First Nations Oral Health Strategy *Teeth for Life*, the Inuit Oral Health Action Plan *Healthy Teeth, Healthy Lives*, British Colombia’s First Nations and Aboriginal Oral Health Strategy *Healthy Smiles for Life*, *New South Wales Aboriginal Oral Health Plan 2014–2020*, and *Filling the Gap* by the Royal Flying Doctor Service [26–33].

According to recent scoping reviews conducted by a group of researchers in Canada, several programs on the integration of oral health into primary care have been developed worldwide during the last decade [34, 35]. These programs have been successful in implementing integrated primary oral health care in terms of reducing patient non-attendance, improving providers’ and patients’ satisfaction, dental visits, screening and prevention of oral diseases, referrals, and access to dental care [34, 35].

However, it is unclear whether successful outcomes of integrated primary oral health care can be applied to Indigenous communities in the same way. Moreover, for effective integrated primary oral health outcomes in these communities, more exploration in Indigenous contexts is required. Hence, this scoping review was guided by the Indigenous concept “two-eyed seeing” (*Etuaptmumk*), developed by Mi’kmaq Elders Murdena and Albert Marshall [36, 37]. It is defined as *to see from one eye with the strengths of Indigenous ways of knowing, and to see from the other eye with the strengths of Western ways of knowing, and to use both of these eyes together* [36]. This approach help in a better understanding of the integrated oral health care in Indigenous communities while valuing both Indigenous and Western knowledge [36, 37]. The objective of this scoping review was to systematically map the available programs and their outcomes on the integrated primary oral health care programs in Indigenous communities underpinned by the two-eyed seeing concept.

## Methods

The methodology of this scoping review was adapted from the five-stage framework by Arksey and O’Malley with the additional sixth stage introduced by Levac et al. [38, 39]. The methodology also drew on the Joanna Briggs Institute’s methodology for scoping reviews, to improve the rigour of the review process [40]. The six stages followed in conducting this scoping review were: 1) identifying the research question, 2) identifying relevant studies, 3) selecting studies, 4) charting the data, 5) collating, summarizing, and reporting the results, and 6) consultation with relevant stakeholders [38–40].

### Identifying the research question

After consulting with the research team members, the following research questions were determined to guide this scoping review:

1. What types of integrated primary oral health care program have been developed worldwide to address the need of Indigenous communities? 2. What were the approaches and outcomes of these programs from the two-eyed seeing concept?

### Identifying relevant studies

The search strategy was developed with the help of an academic librarian at the University of Montreal. The eligibility criteria for the search strategy were developed according to the PCC (Population-Concept-Context), as described by the Joanna Briggs Institute [40]. PCC for this review included publications on integrated primary oral health care services involving Indigenous populations from all around the world, irrespective of study design. Any study protocols, abstracts, opinions, editorials, letters, or commentaries were excluded from the review.

The three-step search strategy recommended by the Joanna Briggs Institute was followed. The initial limited search was performed on Ovid Medline and analyzed text words in the title, abstract, and keywords of the retrieved articles and refined the key terms (Additional file 1) [35]. Then, the second search was conducted using refined key terms across all the included databases such as Ovid EMBASE, EBSCO CINAHL, ProQuest Central, Google Scholar, and Indigenous databases such as the Indigenous studies portal research tool (iPortal), National Collaborating Centre for Aboriginal Health, Native Health Database, and Bibliography of Native North Americans. We used the filters developed by the University of Alberta to retrieve studies related to Indigenous people from OVID Medline, Ovid EMBASE, and EBSCO CINAHL [41]. The third search step looked for journals focused specifically on Indigenous health but not indexed in the databases mentioned above, such as the International Journal of Indigenous Health, Indigenous Policy Journal, and Journal of Indigenous Well-being. This step was facilitated by a manual search of the reference lists of the selected articles to identify grey literature, and by searching for the websites of relevant Indigenous health organizations.

### Selecting studies

The study selection process consisted of two levels of screening. In the first level, one reviewer screened the title and abstract of all retrieved citations for inclusion based on the eligibility criteria mentioned above. In case of any uncertainties, the citations were considered for the second level. At the second level, two reviewers independently performed a full-text review to determine the eligibility of citations. Any disagreements between the reviewers were resolved via discussion to reach consensus.

### Charting the data

A data charting form was developed by the research team to extract relevant study characteristics from selected articles and reports. The following data characteristics were extracted:

- Descriptive study information (authors, year, title, citation, and country).
- Further information depends on whether the article includes an integrated oral health program description or a program evaluation (*program descriptions* [type, program strategy, oral health care provision, outcomes if available]; *program evaluations* [study setting, objective, data collection, indicators, outcomes]).

### Collating, summarizing, and reporting the results

To synthesize and summarize the results, we used the numeral summary of included studies [39] and performed content analysis using a qualitative descriptive approach [42]. With the help of conventional qualitative content analysis, initial codes were inductively generated from the data through an iterative process and then were grouped into categories. The coded material was cross-checked by a second researcher and minor changes were made based upon discussion. Potential program characteristics, approaches, and outcomes for integrated primary oral health care services were synthesized and mapped from all included publications. Then, the programs were categorized based on the types and extent of dental services.

Afterward, the two-eyed seeing concept was incorporated to interpret and synthesize available evidence on approaches for the integration of oral health into primary health care by “weaving back and forth” between Western/biomedical and Indigenous worldviews [36, 43]. The following definitions of Indigenous and biomedical knowledge guided in identifying the respective approaches. *Indigenous knowledge is community specific as it has developed and evolved over time within a specific and localized context through lived experiences, observations, holistic investigative and problem-solving processes. It has been conveyed orally, symbolically, or through experience, and was embedded in the cumulative experience and teachings of Indigenous people* [44]. By contrast, *biomedical knowledge is based on the principle of positivist inquiry, which places value on knowledge gathered empirically through scientific inquiry and assumes that there is a single truth to be discovered* [44]. Furthermore, both the biomedical eye view and the Indigenous eye view were used to assess the outcome measuring instruments and outcome variables. All the outcomes were then divided into four dimensions: biological, mental, emotional, and spiritual. The use of Atlas.ti software facilitated the analysis. This scoping review follows the PRISMA Extension for Scoping Reviews (PRISMA-ScR) [45] guideline for reporting the manuscript.

### Consultation with relevant stakeholders

Consulting with stakeholders enriches the comprehensiveness of the review as well as facilitating wider knowledge transfer. For this scoping review, we included academic health care professionals and Indigenous community partners in the research team to consult on the research questions and the search strategy, and to provide input on the data analysis.

## Results

**Fig. 1:**
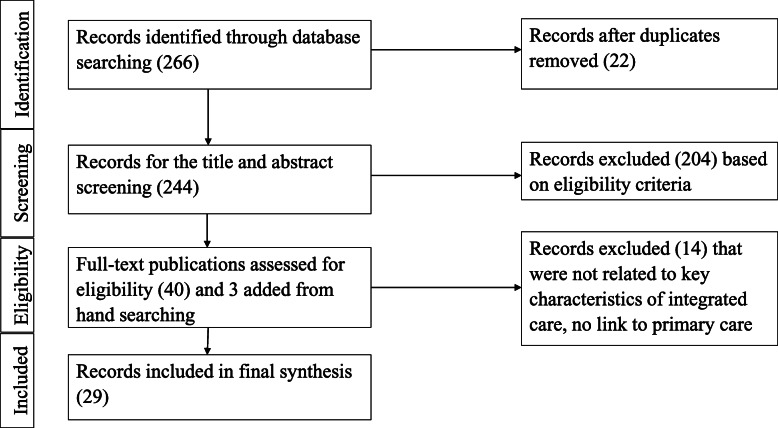
Flow of study selection

Figure 1 outlines the search strategy and article selection. The final search strategy extracted 266 records. After removal of duplicates, a total of 244 publications were screened by title and abstract and 204 publications were excluded as they did not meet the inclusion criteria. A total of 40 publications were screened during full-text review; three relevant articles were added after hand searching the references of the available publications, and 14 publications were excluded. In the end, 29 publications describing 30 programs on integration of oral health into primary care from 1972 to 2019 were included in this scoping review. The selected publications consisted of 15 articles describing primary research (12 quantitative studies, one qualitative study, and two mixed-methods studies), eight original field reports and case studies, five publications describing the application of the framework to the integration of oral health in primary health care in the form of an annual report or manual, and one literature review [25, 46–73]. The quantitative studies included a wide variety of study designs, including cluster randomized trial, non-experimental trial, community trial, intervention study, retrospective study, cross-sectional, and pilot study [48, 50, 54–56, 58–60, 62–64, 73]. These programs were conducted in Australia, the USA, and Canada, mostly in the last two decades. Tables 1 and 2 outline the characteristics of the included programs and program evaluations respectively.

**Table 1:** Characteristics of included programs on integrated primary oral health care in Indigenous communities

Author, year and country	Type of program	Program strategy	Oral health care provision
Bain and Goldthorpe, 1972, Canada [46]	University of Toronto’s Sioux Lookout Project	• Collaborative services for Cree and Ojibway people in Sioux Lookout region involving multidisciplinary health services including dental services	• Development of dental clinic at one nursing station • Basic dental facilities in all nursing stations and some satellites • Dental care provided by dentists and interns • Development of caries prevention program **Outcomes:** Program was found to be feasible after 3 years.
Chiarchiaro G, 1997, USA [47]	Indian Health Service in Oklahoma	• Oklahoma dental program as part of integrated health care system	• Clinic-based dental services and community-based oral health promotive and preventive services • Population based program such as for school children, people with special needs • Multidisciplinary team working with cleft palate team
Lawrence HP, 2004, Canada [48]	Community-based dental-hygiene coordinated Prenatal Nutrition Program	• Community-based dental preventive program for Early childhood caries at Sioux Lookout Zone performed by Woman and Child Community Nutrition Program workers • Cross-sectional survey was conducted among 2 to 5-year old Anishnaabe children from 16 communities where 8–8 communities were identified as high-low intervention communities based on frequency and coverage of health worker and participants contact	• Trained health workers provided culturally sensitive nutrition and dental preventive education to pregnant women, new mothers, and elders raising children during home visits • Promotion of healthy food, optimal oral hygiene practices via head start brushing • Reinforcement of healthy dental care practices by nurses during Well Child Clinics • Offered oral educational packages to caregivers • Media campaigns and distribution of posters and pamphlets • Smoking cessation sessions ***Outcomes:*** • Overall positive outcomes • Improved hygiene and reduced number of decayed surfaces • Improved oral health knowledge and preventive practices of caregivers and less untreated carious teeth significantly higher among the high-intervention communities • But still higher demand for dental services under GA
Parker EJ et al., 2005, Australia [49]	Oral health program by Pika Wiya Health Service Inc.	• Implementation of the first phase of the program in 2001 to develop culturally relevant quality oral health care services	• Provision of dental services for eligible adults 2 days per week • Oral health promotion at schools’ festivals and Pika Wiya health service open days. • Referral for patient transport services to radiology department and local pharmacy ***Outcomes:*** • Program was successful • More satisfied community members
Harrison RL et al., 2006, Canada [50]	Brighter Smiles	• This participatory research program aimed to improve children’s oral health in a Hartley Bay First Nations community by providing service-learning experience to paediatric residents	• Oral health care provision via classroom teachings, school-based brushing, fluoride application, and regular visits by UBC paediatric residents for well-child care. ***Outcomes:*** • Service-learning experience was successful • More preventive treatments were offered compared to restorative or rehabilitative treatments
Kruger et al., 2010, Australia [51]	Case study	• To discuss 10 years experiences of Centre for Rural and Remote Oral Health in Western Australia	• Oral health services via developing vertically integrated service, education and research driven model • Co-location • Provision of Fly-in and fly-out services • Symbiotic relationship among health and dental care providers that creates a supporting environment • Interprofessional collaboration • Culturally relevant services by involving IHW • Interprofessional education
Meihubers S, 2013, Australia [52]	Bila Muuji Oral Health Promotion Partnership Program	• Bila Muuji Aboriginal Health Service initiated this program involving primary care workers at Aboriginal Community Controlled Health Organisations • Target groups: children less than 5 years, school-aged children, young adults, people with chronic disease, and the elderly	• Appointment of oral health promotion coordinator • Oral health promotion programs including school-based daily toothbrushing, oral health information sessions; training primary care staff. ***Outcomes:*** • Continuing • Positively accepted by the community • Improved oral health profiles
Mathu-Muju KR, 2016, Canada [54]	Children’s Oral Health Initiative (COHI) – Community-based preventive program for First Nations and Inuit children	• Its short-term aim was increase access to preventive oral health care services and long-term outcome to decrease levels of dental disease. • Conducted by Dental hygienists and therapists under Health Canada for Aboriginal communities and COHI aide (trained community health workers) • Target groups: preschool children, school children, parents/primary caregivers, and pregnant women	• Preventive oral health care services included fluoride varnish, pit and fissure sealants, oral health counselling and atraumatic restorative therapy. • COHI aide helps in explaining program purpose to parents and obtains inform consent, oral health education, schedule dental appointment as well as fluoride varnish application. ***Outcomes:*** • Program has been successful • It extended to 320 communities over a period of 10 years from 2004 to 2014 along with increase in number of participating children.
Wooley S, 2016, Australia [65]	Nganampa Health Council Dental Program	• Commenced in 1986 to provide accessible, appropriate and effective oral health	• Oral health care via 2 dental operatories at health clinics, mobile dental units (dental truck) and portable dental equipments • Oral health services included promotive services, emergency Service, school dental program, adult dental program, special needs, and prosthodontics. ***Outcomes:*** • 100% rate of sealants and varnishes in children. • More dental caries in children at 6 years, lesser DMFT in 12 years children and more restorative unmet needs, diabetes associated periodontal diseases and edentulousness in adults, • Still challenges were existing and necessitates further coordination and referral programs.
Maari Ma Health Aboriginal Corporation, 2016, Australia [66]	Evaluation of Maari Ma Health Aboriginal Corporation’s Chronic Disease Strategy	• Integration of oral health into health programs ‘Healthy start’ and ‘Keeping well program’ • ‘Clean Teeth Wicked Smiles’ oral health promotion program for school-aged children • ‘Tiddilicks’ program for pre-school children promoting tooth brushing and drinking water rather than fizzy drinks • Incorporation of oral health into health screening, fluoride varnish and fissure sealants application • Fluoride varnish application as part of GP’s child health checks • ‘Filling the Gap program’: volunteer dentists visit for 1–2 week period	• Promotion and prevention of oral health via public health dentist, dental therapist and Indigenous dental assistants • Treatment done by dentist ***Outcomes:*** • ‘Clean Teeth Wicked Smiles’- significant increase in the number of children brushing twice or more a day • Significant reduction in decayed primary and permanent teeth in children
Cree Board of Health and Social Services of James Bay, 2004, Canada [67]	Cree Board of Health and Social Services of James Bay developed the Strategic Regional Plan 2004–2014	• Implemented an integrated delivery of health and social services in the Cree communities including integration of oral health into primary care	• CBHSSJB has developed a separate dental clinic in each Community Wellness Centre in all communities • Provision of free services by dentists and dental hygienists • Preventive oral health programs carried out by various dental and non-dental healthcare providers
Torres and Cape Hospital and Health Service, 2018, Australia [68]	The Torres Strait Primary Oral Health Care Project 2017–2019	• Aimed to develop oral health roles for remote primary health providers by integrating oral health assessments and first response duties within remote Primary Health Care Centres via telehealth technologies	• Use of videoconferencing by the dental team at Thursday Island hospital to train and support health care providers at rural and remote primary health care centres in their oral health activities as well to facilitate communication and clinical consultations among them ***Outcomes:*** • Program’s implementation phase resulted in improved oral health assessment and promotion by primary care providers
Ontario’s Aboriginal Health Access Centres [69]	Waasegiizhig Nanaandawe’iyewigamig	• Provision of comprehensive primary health care services to its first nations communities including travelling health care providers to remote areas	• Health promoters with dental hygienist include offer promotive and preventive oral health services including via COHI
Ts’ewulhtun health centre annual report 2017–18 [72]	Ts’ewulhtun health centre, BC, Canada	• Offer primary health services to Cowichan tribes	• Co-located dental clinic • Offer oral health education, prevention and restorative treatment to all community members including COHI • Oral health promotion within public health programs
Sts’ailes primary health care project: report, 2013 [25]	Nisga’a Valley Health Authority (New Aiyansh, BC)	• Offers primary health services to Nisga’a communities	• Co-located dental clinic
Sts’ailes primary health care project: report, 2013 [25]	Anishnawbe Health (Toronto, ON)	• Offers primary Health Care Services by a multidisciplinary team, including dentist	• Offers promotive, preventive and clinical dental services • Delivers Healthy Smiles Ontario Program
Southcentral foundation’s Nuka System of Care [25, 72]	Alaska Native and American Indian people in the Anchorage Service Unit area	• Integrated primary health care model with wide range of health services including dental services	• All types of dental services
Indian health services [71]	Federal health services to American Indians and Alaska Natives	• Integrated dental services with other health care services	• Ranges from basic and prevention services to all dental treatments

**Table 2:** Characteristics of included program evaluations on Integrated primary oral health care in Indigenous communities

Author, year and country	Type of Study	Study objective	Setting	Data collection	Indicators	Study outcomes
Pacza T, et al. 2001, Australia [55]	Pilot study	• To develop IHW training program with the proper teaching methodologies assuring its effective delivery and to assess students’ experience	• Pilot training program developed as a prerequisite to a culturally appropriate preventive oral health program • Conducted as series of modules at two Indigenous training schools	• Observation • Questionnaires	• Program effectiveness • Students’ feedback	• Program was effective and identified considering 10 students per trainer • Students were satisfied and considered this training relevant to their needs.
Macnab AJ, et al., 2008, Canada [56]	Intervention Cross-sectional study	• To improve oral health and oral health knowledge among school children	• Community visits by a team of 2 trained medical residents with one supervisor • Integration of oral health program with well-baby and well-child clinic • Incorporation of regular toothbrushing sessions, fluoride rinse and varnish application and dental health anticipatory guidance and classroom presentation by residents	• Pre-post intervention examination by dentist • Community feedback	• dmfs/DMFS • Caries free status • Questionnaire on oral habits • Subjective community experience	• dmfs/DMFS measures improved, and caries free children increased from 8 to 32% after 3 years of intervention • Improved oral health behaviours • Community responded positively for the program.
Jackson-Pulver L, et al., 2010, Australia [57]	Program evaluation/ Mixed method	• To develop a ‘Filling the Gap’ - volunteer dental program in partnership with the local community controlled primary health service	• Wuchopperen Health Service integrated dental services via a base clinic and mobile dental clinic • Provision of visiting volunteer dentists	• Literature review • Quantitative using patient health records and • Qualitative using semi-structured interviews	• Episodes and type of care • Effect on waitlist • Stakeholders’ perception about the program	• Increased episodes of dental care and enrolment of new patient as well as increased volunteers’ visits. • Meeting patient needs and reducing waiting list • Improved workforce development and care continuity
Dyson K, et al. 2012, Australia [58]	Retrospective study	• To examine the cost-effectiveness of networked hub and spoke visiting model of Indigenous rural oral health services	• Integration of dental clinic with Indigenous health services at 5 rural sites	• Financial analysis (Measurement of service provision)	• Costs to value of care ratio (data retrieved records for the years 2006, 2008 and 2010)	• Cost to value ratio was 1.61. • No significant different among 5 sites • Cost to value ratio is similar to Government estimates (1.5–2).
Parker EJ et al., 2012, (Aboriginal Children’s Dental Program in Port Augusta) Australia [59]	Intervention study/ Evaluation after 3.5 years	• To provide a cultural-friendly dental service	• Dental services by IHW and dentists, also in collaboration with dietician • IHW were trained via dental students at Adelaide’s dental school through workshop	• Oral health related hospital records • Informal interviews with health service staff	• Services statistics • Key issues and challenges in the program	• Improved participation rates, increased number of preventive treatments compared to restorative treatments • Key issues and challenges: issues related to consent, cancelled and failed appointments, difficulty in contacting and communicating parents and guardians
Harrison RL et al., 2012, Canada [60]	Cluster-randomized pragmatic trial	• To compare the dental health status of young Cree children whose mothers received maternal counselling with that of children whose mothers only received educational pamphlets	• Oral health related Motivational interview-style counselling by trained community health representatives or local women in test communities • Distribution of educational pamphlets to mothers	• Dental examination • Questionnaire	• Dental caries assessment (Pitts criteria) at 30 months of age • Mothers’ dental health knowledge, behaviour and child caries related quality of life	• Low caries prevalence in test group compared to control, but not statistically significant. • No significant difference for maternal oral health behaviours and child quality of life.
Portland District Health, Winda-Mara Aboriginal Corporation, 2012, Australia [73]	*Deadly Teeth:* promoting oral health in Gunditjmara country	• To provide a culturally appropriate oral health promotion services	• Oral health promotion services for families with children up to 5 years old • Distribution of tip card including eat well, drink well and clean well tip cards	• Pre- and post- survey questionnaire over phone	• Culture appropriateness of the program	• 100% services believed that services were culturally appropriate.
Willder S et al., 2014, Australia [61]	*‘Indigie-Grins’ program*- A community-based oral health promotion program- Mixed method study	• To assess the oral health status of Indigenous children aged 5–12 years • To develop and provide a culturally appropriate community intervention program	• IHWs helped in recruitment, retaining and education of the children and families during research • They also participated as the principal researcher and designed the culturally specific aid and equipment for oral health promotion	• Oral health assessment by using dental caries and periodontal health indices • Focus group discussion	• Oral health status • Participants’ perception and attitude towards oral health (both pre- and post-)	• Improvement in unmet restorative needs, improved periodontal status of children • Improved access, awareness and oral health behaviours of children and parents
Braun PA, et al., 2016, USA [62]	3-year Cluster-randomized community-based trial	• To measure the effectiveness of the program in reducing the caries increment in head start attending Navajo children	• Interventions (oral health promotion and Fluoride varnish application) were provided by trained Indigenous paraprofessionals, named as *community oral health specialists*.	• Oral examination, questionnaires	• Primary outcome indicator: change in dmfs with time • Secondary outcomes indicators: DMFS, caries prevalence, caregiver oral health knowledge and behaviour	• No difference in caries reduction among intervention and control groups • Improved knowledge among care giver at 1 year (but not at 2 and 3 year)
Murphy KL, et al., 2017, USA [63]	Non-experimental quality improvement project	• To integrate and evaluate a pediatric oral health project in an American Indian pediatric primary care setting	• This study involved pediatric and dental clinic at an Indian Health Service hospital • Primary care providers had completed *Smile for Life* Curriculum • They performed oral health screening, caries risk assessment, oral health education for parents and caregivers, and dental home referral	• Oral health screening and carried risk assessment using oral health risk assessment tool	• Oral health assessment • Dental referrals	• Around 91% children assessed having high caries risk • 72.4% referral and 74% of these were seen by the dentist
Mathu-Muju KR, 2017, Canada [53]	Qualitative research	• To explore the experiences of First Nations families whose children had enrolled in the COHI program	• COHI – Community-based preventive program for First Nations and Inuit children	• Semi-structured interviews	• Perception of community members whose children participated	• Improved oral health knowledge and behaviour of children and caregivers • Improved access to preventive and restorative services • Promoted continuity of care that facilitated referral and linkages for oral health care
Smith L, et al., 2018, Australia [64]	Community trial	• To evaluate the effectiveness of a dental health education program, *Smiles not Tears,* in preventing Early Childhood Caries in Indigenous children	• IHWs delivered age appropriate oral health education to families over five visits, screened children and distributed culturally appropriate resources • At 6th visit, dental examination was done by dentist	• Dental caries indices (dmft, dmfs, Sic10 and SiC30)	• Comparison of caries prevalence of children at 30 months of age with children in control group	• More children in test group were caries-free compared to control group

### Characteristics of programs

The following four categories of programs emerged from the analysis and represented the synthesis of the data: oral health promotion and prevention programs (*n* = 13), comprehensive dental services (*n* = 13), fly in, fly out dental services (*n* = 3), and teledentistry (*n* = 1).

#### Oral health promotion and prevention programs

Most of the programs were identified in this category. These programs were essentially targeted at Indigenous children, parents, and caregivers [48, 50, 53, 54, 56, 60–64, 73]. However, the target populations for a few programs involved pregnant women, young adults, people with chronic disease, and the elderly [48, 52, 55, 73]. The strategies for oral health promotion and prevention included culturally relevant oral health education, distribution of oral health aids and equipment, maternal counselling during pregnancy, and pediatric visits to community hospitals and schools [48, 56, 60, 62–64].

#### Comprehensive dental services

Various Indigenous health services in North America and Australia have integrated oral health into primary care for all age groups via incorporating dental clinics with health care services, providing basic, emergency, specialist, and referral services, and developing oral health prevention and promotion programs [25, 47, 49, 59, 65–67, 69–72]. Bain et al. described feasible integrated oral health care services in all nursing stations and associated satellite clinics for Indigenous people of the Sioux Lookout zone in Canada [46].

#### Fly in, fly out dental services

In these services, health professionals work in remote and inaccessible areas by flying there temporarily. Dyson et al. and Jackson Pulver et al. reported a networked spoke-and-hub model of visiting services and volunteer visiting dentist programs, respectively, in the integrated primary oral health care set-up for Indigenous communities in Australia [51, 57, 58].

#### Teledentistry

The Torres and Cape Hospital and Health Service in Australia has integrated dental telehealth consultation for its rural and remote primary health centers [68].

### Approaches for integrating oral health services within primary health services

A variety of approaches have been used to integrate oral health within primary care services, drawing from biomedical as well as Indigenous worldviews. Table 3 outlines the strategies used in implementing biomedical and Indigenous approaches to integrate oral health into primary health care.

**Table 3:** Strategies used in implementing Western and Indigenous approaches to integrate oral health into primary health care

Western Approaches	• Development of one integrated dental clinic at one satellite centre for comprehensive dental services, provision of basic dental services at other nursing stations and satellite centres [46] • Basic or Comprehensive dental services [25, 47, 49, 59, 63, 65–67, 70–72] • Community based promotive and preventive dental services (community water fluoridation, school based programs, pit and fissure sealants, fluoride varnish, education, tobacco counselling, maternal counselling prenatal and well baby visits) [25, 47–49, 52–56, 59, 61–63, 65–67, 69, 70, 73] • Mobile dental services [57] • E-oral health by rural primary care providers (teleconferencing) [68] • Visiting dentists [57] • Training of non-dental primary are providers on dental health [50–52, 54, 55, 58, 60, 63, 64]
Indigenous Approaches	• Aimed for culturally competent services [46] • Community ownership and partnerships with Indigenous communities [48–54, 56, 58–62, 64–66, 71–73] • Culturally appropriate oral health service such as development of dental education tools in native languages [48, 67], Oral health promotion by interconnecting with the community at their local and cultural events such as circle of wellness program [70], use of Indigenously adapted aids and equipment such as toothbrushes, timers and brushing charts [61], role of elders and family tip cards designed by Indigenous Families and painted by Indigenous artist [73] • Locally trained IHWs [25, 48, 49, 51–55, 58, 59, 61, 62, 65–67, 69, 73] • Cultural training/advice for non-Indigenous health care providers [59, 73]

#### Biomedical approaches

These approaches to integrating oral health into primary health care include leadership and governance, administration and funding, capacity building, infrastructure and technology, team work and coordination, and evidence-based practice**.**

##### Leadership and governance

Most of these programs were governed by the collaboration of federal and provincial government agencies, Indigenous health organizations, national health research councils, and academic universities [46, 47, 52–54, 59, 65, 67, 73]. In one program, a non-profit organization also collaborated [48]. These programs were focused on accomplishing aims and objectives mentioned in their action plans, strategic plans, or policy papers [46, 49, 52]. In addition to providing quality service delivery, they also concentrated on providing continuing education and training for dental and non-dental staff [47, 50–52, 54, 55, 58, 60, 63, 64].

##### Funding and administration

Many programs were financially and administratively supported by governments, Indigenous organizations, and universities [46, 49, 50, 52, 54, 59, 61, 64, 66]. They have developed funding models for facilitating access to oral health services for these communities. These programs highlighted the importance of continued funding for program sustainability [54, 59, 64]. For instance, the lack of continuous financial support adversely affected care continuity in the Aboriginal Children’s Dental Program as it resulted in the withdrawal of an Indigenous health worker’s (IHWs) job position [59].

Other examples for administration and funding included the provision of free basic oral health services to patients [58, 66, 67] and administrative support for travel and accommodation in the case of visiting specialists [66, 67]. Pika Wiya Health Services also provided transport services for eligible clients for X-rays and medicine [49], and *to and fro* transport services between school and dental clinic for children and parents. In the Maari ma region, oral health services are provided by dentists, dental assistants, and trained Indigenous dental assistants, but if there are no dentists then fly in, fly out services, or volunteer dentist services are arranged [66]. These administrative supports enhanced access, care continuity, as well as coordination among professionals [58, 59, 66, 67].

Moreover, many organizations incorporated preventive oral health programs into public health programs such as the Community Nutrition Program, well-baby program, maternal and child health program, healthy start program, or the chronic disease program, to enable increased accessibility for outreach oral health services [48, 52, 65–67, 70].

##### Capacity building

Various programs emphasize developing training sessions for non-dental health professionals and IHWs [50–52, 54, 55, 58, 60, 63, 64]. In one program, primary health care providers were trained for oral health care assessment [63]. In some cases, these professionals successfully completed the *Smile for Life* curriculum, which is focused on providing educational resources for integrating primary oral health care [63]. This program also suggested that integrated oral health services also facilitate inter-professional collaboration [63]. Similarly, in the *Brighter Smiles* program, pediatric residents were trained by attending at least 1 day with dental staff at the Children’s Hospital, as well as academic sessions on dental health topics [50]. Interprofessional dental training also helped in making non-dental primary care providers aware of their role in improving people’s oral health [50].

##### Infrastructure and technology

These programs have developed an infrastructure that supports integrated primary dental services [46]. For instance, several programs made provision for a dental clinic with primary health service facilities [25, 46, 49, 57–59, 63, 65, 67, 70, 71]. This facilitated accessible integrated care in these programs. Likewise, in the Sioux Lookout project, a dental clinic was developed at one nursing station, and at least basic dental facilities were provided at all nursing stations and some satellite centres [46]. The Indian Health Services in the USA has developed hospital-based and ambulatory health care centre based dental clinics [47].

Similarly, the use of technology in the form of shared electronic health records helps in coordinating and facilitating the wide range of information for patient follow-ups and referrals, access, cost, productivity, as well as quality assurance [65, 71]. Examples include the *Internet-Based Electronic Patient Management System – Communicare* at Nganampa Health Council Dental Program, which utilizes shared electronic health records, and the *Resource and Patient Management System* at Indian Health Services in the US. In addition, some organizations are working on e-oral health technology, especially to integrate tele-dental consultations for their rural and remote primary health centres [68].

##### Team work and coordination

These programs involved effective team work and interprofessional coordination among dental care providers (dentist, dental hygienist, dental therapist) and other health workers including IHWs, nutritionists, pediatricians, other clinical staff, and teachers in the case of school services, as well as administrators [48–52, 54, 56–59, 61, 63–66]. For instance, the Maari ma program operated with a vision of providing a coordinated family-based approach through an integrated multidisciplinary team of health care providers [66].

Oral health assessment by non-dental primary health care providers facilitated interdisciplinary coordination and referral services [63]. Some programs appointed a manager, liaison officer, or regional coordinator for facilitating coordination and management of oral health services and linking all stakeholders [46, 52, 60, 64].

Smooth coordination and cooperation among personnel from multiple organizations are vital for the program’s success. For example, a funding model for the Aboriginal child dental program at Pika Wiya Health Services was successful due to proper coordination among three organizations, namely, South Australian Dental Service, Pika Wiya Health Services, and the Spencer Gulf Rural Health School [59].

##### Evidence-based practice

The biomedical world is scientific and believes in only one truth relying on scientific laws. By contrast, the Indigenous worldview relies on beliefs and a spiritual world and can include multiple truths based upon individual experiences. The “biomedical eye” view for this project suggested that the selected programs were implemented and evaluated based on evidence-based literature [52, 54, 59, 61]. Implementation of these programs was decided based on evidence of poor oral health knowledge, oral health status, inadequate access to dental services, and need for such primary oral health care services [54, 67, 73]. Furthermore, their execution was also influenced by evidence of integrated good quality and culturally adapted primary health and oral health care services in improving Indigenous health and oral health status [59, 74].

#### Indigenous approaches

Indigenous approaches to integrated oral health into primary health care were categorized as: vision for holistic health, culturally appropriate services, community engagement, shared responsibility and partnership, and cultural competence.

##### Vision for holistic health

Most of these programs in Indigenous communities have a vision and mission to achieve holistic health wellness by working together in a multidisciplinary health care model [25, 52, 61, 65–73]. This concept of holistic health is highlighted, with a focus on biological, mental, emotional, and spiritual wellnesses of individuals, families, and communities at a larger scale.

##### Culturally appropriate services

Along with a vision to provide holistic health, incorporating cultural values and beliefs was considered necessary in most programs [25, 52, 61, 65–70, 72]. Moreover, some Indigenous health organizations consider the culture to be at the centre of all health care activities and acknowledge “culture as treatment” in tackling health and oral health problems among Indigenous populations [67, 69].

##### Community engagement, shared responsibility, and partnership

Community ownership and community-based partnerships were considered essential elements for these programs’ success [48–54, 58, 59, 61, 62, 64–66, 72]. Indigenous communities should give their consent to participate in the program [53, 54]. From the beginning of program design, they were involved in managing and making decisions about the ongoing programs [53, 54, 59]. These approaches emphasize the development of culturally relevant programs by Indigenous people for Indigenous people [25]. Also, these approaches have been associated with the sustainability of such programs and positive health outcomes [25].

##### Cultural competence

Community capacity building is one of the guiding principles for developing partnerships with Indigenous populations. It involved trained local community health workers who facilitated the provision of culturally appropriate services [49, 53, 58, 59, 62] and consequently improved program participation and acceptance by community members [53, 59]. These local health workers act as a link between primary health care providers and community members [51, 54, 57–59, 65].

In most cases, the trained IHWs participated in delivering oral health education and counselling for children and families [48, 53, 54, 60–62, 64], helped in children’s oral examination by lifting the lip [64], designed and distributed culturally specific oral health promotion aids [61, 64], scheduled dental appointments [53, 54], and applied fluoride varnishes [53, 54]. In some cases, they visited participants’ homes for oral health education [54, 61, 64].

### Program outcomes

As per two-eyed seeing guiding principles of Indigenous research, the program outcomes are described from biomedical and Indigenous perceptions. Among all selected programs, a few were focused on reducing early childhood caries [53, 54, 60, 62–64]; however, others aimed to improve overall oral health status and oral health knowledge of children [48, 50, 56, 59, 61].

### Outcomes from Western biomedical approaches and indigenous approaches

We found that the selected studies intended to report their outcomes considering biomedical approaches and did not take into account the holistic outcome variables from Indigenous approaches. For these outcome measurements, data were collected via questionnaires, oral health screening and assessment, observation, interviews, patient records, online surveys, and financial analysis. The outcome variables included change in oral health status, oral health knowledge, attitude, and behaviour of Indigenous participants, perception of care providers, change in types of dental services, and cost-effectiveness.

In assessing these available outcomes from the Indigenous eye, the outcomes were divided into four dimensions, namely, biological, mental, emotional, and spiritual. The biological (dental health-related) dimension included outcomes related to change in dental status, types of services, and accessibility of these services. The mental dimension corresponded to change in oral health knowledge, and attitude of Indigenous participants and care providers, whereas the emotional dimension reported changes in behaviour and perceptions around primary oral health care. However, this review did not identify any study measuring the spiritual dimensions of primary oral health care services.

#### Biological (dental health-related) outcomes

Overall, the programs were found to be effective and feasible [46, 48–50, 54, 59]. The selected integrated oral health programs improved oral health care access and oral health status of Indigenous children and parents [48, 52, 56, 61, 64, 66]. These programs improved preventive treatments compared to restorative or rehabilitative treatments for children [50, 59]. They also resulted in increased children’s oral health assessment, preventive services, and referral services by primary care providers [59, 63, 65, 68]. However, one program demanded more coordinated and referral programs to combat persisting dental caries, periodontal problems, and edentulousness [65].

Furthermore, two cluster randomized control trials of oral health promotion interventions on maternal counselling and oral health promotion found fewer caries among children in test groups. Nonetheless, these trials did not find a significant difference among test and control groups in relation to children’s caries prevalence and caregivers’ oral health behaviour [60, 62]. However, explanatory analysis for these trials reported better treatment effect with increased numbers of maternal counselling interventions and recommended the use of culturally appropriate interventions to reduce severe dental caries in Indigenous children [60, 62].

Provision of visiting dentist services in remote areas improved access to dental care, reduced waiting lists, and met communities’ oral health needs [57]. The visiting dentist program was also recognized to be effective by administrators, care providers, and patients in terms of addressing community oral health needs, offering continued services, and improving the availability of health workforce [57]. Dyson et al. reported on a networked spokes-to-hub model of visiting services as cost-efficient in delivering oral health care in rural Indigenous areas [58].

#### Mental outcomes

These program results suggested improved perceived oral health knowledge and behaviour among children and their caregivers as well as improved access to oral health care [53, 61].

#### Emotional outcomes

Most of the programs were well accepted by the Indigenous communities and reported satisfaction among community members [49, 52, 55, 56]. Interprofessional training programs for Indigenous primary health providers were effective and appreciated by these trainees [55].

## Discussion

The evidence from this study implies that integrated primary oral health care programs can improve Indigenous oral health-related outcomes. Integrated primary health care has the potential to combat health and oral health care disparities among Indigenous populations, as integrated care offers coordinated care for multimorbid conditions prevalent in Indigenous communities [75, 76]. The concept of primary health care comprises a holistic view of health that goes beyond the narrow biomedical model and includes biomedical, psychological, and social dimensions of health and wellbeing [23, 77]. It is conceptualized as person-focused and population-based care [78, 79]. Likewise, integrated care also incorporates a biopsychosocial and spiritual model of health care services and focuses on patient-centred care [75, 76].

Among the various approaches available for literature reviewing, we found the scoping review most suitable in performing this study, considering the aim mentioned. The scoping review allowed broad and thorough mapping of the available literature on the integration of oral health into primary care in the Indigenous context, irrespective of quality of the literature [38, 40]. Furthermore, the use of two-eyed seeing as a guiding principle in the scoping study facilitated a thorough analysis of the data and acknowledgment of Indigenous knowledge and culture and their impacts [43]. This approach has the ability to better recognize the health challenges in Indigenous populations [80]. Two-eyed seeing offered a platform to non-Indigenous researchers by providing them the opportunity to understand traditional knowledge and culture [81].

**Fig. 2:**
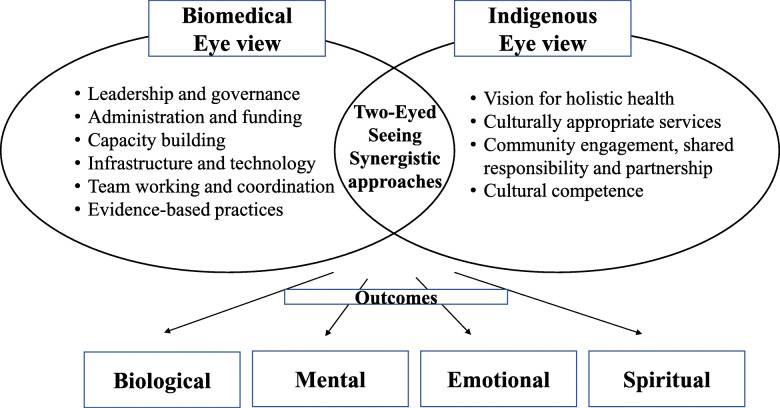
Flowchart illustrating two-eyed seeing view of approaches and outcomes of programs on integrated primary oral health services

This scoping review draws together evidence mapping of the types and outcomes of integrated primary oral health care models in Indigenous communities and also identifying the essential approaches for integrating health care services for Indigenous populations in these models. Our results suggest that community-based and culturally appropriate integrated primary oral health care programs were successful in improving oral health status and knowledge of Indigenous communities, especially in rural and remote areas. This review identified four types of programs for integrated primary oral health care in Indigenous populations: oral health promotion and prevention programs; comprehensive dental services; fly in, fly out dental services; and teledentistry. The approaches from the biomedical worldview included governance and leadership, administration and funding, capacity building, infrastructure and technology, team work and coordination, and evidence-based practice. Approaches from the Indigenous eye included a vision for holistic health, culturally appropriate services, community engagement, shared responsibility and partnership, and cultural competence. These programs mainly evaluated biological, mental, and physical outcomes, with no measure of spiritual outcomes. Figure 2 illustrates the two-eyed seeing view of approaches and outcomes of programs on integrating oral health services within primary health services. Evidence on approaches and outcomes of integration of oral health into primary care in the general population is consistent with our results [34, 35, 82]. The identified approaches of the integrated primary oral health care interventions extended from the micro to macro level of integration in the form of colocation, interprofessional coordination, interprofessional training and education, integration of oral health into public health programs, financial support, shared health records, cultural safety, and shared vision and mission [78, 79]. Our results also correspond to the most common values of integrated care described by a recent systematic review by Zonneveld et al. [83]. According to Leutz’s concept of levels of integration (linkage, coordination, and full integration), most of the selected programs correspond to the level of linkage and coordination [84]. Programs at the linkage level focused on identifying and managing urgent oral health needs, referral, and follow up [84]. Some programs were identified at the coordination level in terms of smooth service transition, information exchange, suitable financial model, and full population coverage [84].

Various health systems around the world embrace integrated care as a potential way to address the rising demand for better health-related outcomes and patient experiences, specifically for chronic and multimorbid patients [85]. As per previously reported literature review, the characteristics of integrated health care systems include value-driven governance & leadership, hospital/physician alignment, financial integration, clinical integration/care coordination, information continuity, patient-centred & population-health focused, and continuous quality improvement & innovation [86]. Our results are in line with these characteristics, highlighting their influence on the integrated primary oral health care services in Indigenous populations. The available evidence suggests that there cannot be one single model that best supports integrated care. Instead, the integrated health care model can only be successful if it is adapted to the needs and characteristics of the local population [85]. Considering the Indigenous eye view, the provision of culturally appropriate services was identified specific to Indigenous settings. Thus, the characteristics mentioned above offer better integrated services to these settings by incorporating culturally relevant services. Our results also correspond to the eight characteristics of Indigenous primary health care service delivery models identified by Harfield et al.: culture, accessible health services, community participation, continuous quality improvement, flexible approach to care, culturally appropriate and trained workforce, holistic health care, and self-determination and empowerment [16].

The Indigenous primary health care models emphasize the role of culture in health care service provision, in contrast to conventional biomedical models of primary health care that do not specifically signify cultural aspects in care delivery [16]. Strategies to integrating culture included Indigenous communities’ ownership, empowerment, and capacity building, as well as provision of culturally relevant oral health services. Previous studies have also identified the key role of Indigenous communities’ ownership, empowerment, and self-determination in improving their local health services and health outcomes [16, 87, 88]. Indigenous health service organizations such as Aboriginal Community-Controlled Health Organisations or Indian Health Services prioritize working on the principle of Indigenous peoples’ right of ownership and participation [16, 71, 89].

Cultural importance in Indigenous integrated primary oral health care delivery in our results is in line with previous integrated health care services and programs [16, 75, 76]. The role of IHWs strengthened the integration of culture in included programs. However, these workers were given a variety of titles, such as Aboriginal Health Workers, community health representatives, community oral health specialists, Children’s Oral Health Initiative Aides, or strained nutrition educator [48, 52, 53, 58, 60, 61, 64, 67, 89]. Moreover, training of IHWs is a sustainable and cost-effective solution as such training incurs less cost compared to the travel costs required for regularly visiting dentists [64].

In some cases, fly in, fly out services were considered relevant to Indigenous communities with a lack of dental care providers [57, 58]. It is possible that services of this sort may not be coherent with the Indigenous core values [57]. However, the continuous presence of local health providers outweighs the problem of discontinuity associated with fly in, fly out services and visiting dental services [57, 67]. These services are successful in the short term but should not be considered as a permanent solution for improving Indigenous oral health services [57].

The approaches identified in this review were associated with some barriers that adversely affect the integration of primary oral health care. These barriers were: difficult human resource management, administrative barriers, difficult communication, and discontinuity of care. Difficulty in human resource management involved workforce shortage [54], high staff turnover in rural and remote areas [60, 64], and intermittent services due to fly in, fly out staff or visiting staff in rural areas [57]. Administrative barriers included irregular or lacking financial resources [54, 59, 64]. Barriers posed by difficult communication and discontinuity of care were associated with difficulty in contacting patients or caregivers [59] due to frequent change of mobile phone numbers and addresses [64], cultural and traditional *move around* [64]*,* lack of understanding of the importance of dental care [59], and low oral health literacy among community members [59].

### Strengths and limitations

This scoping review, to our knowledge, is the first study to assess integration of oral health into primary health care in Indigenous communities. Another strength is that this is a systematically performed review using a robust methodology that ensures the transparency of the findings. Moreover, this scoping study was Indigenously adapted by applying two-eyed seeing in assessing the evidence.

There were a few limitations in this scoping review. First, the review could not include publications in languages other than English and unpublished data. As well, quality assessment could not be performed due to the nature of this review, as it involved a variety of studies, including program descriptions.

### Study relevance and future research

Our results, nevertheless, may be of interest to Indigenous communities globally that are seeking to improve their health and oral health status. This review contributes to the development and operationalization of the best integrated primary oral health model for Indigenous populations.

Our scoping review reflects the need for more contribution of traditional knowledge and culture in integrating oral health services. Most of the programs aimed to provide at least basic dental services to the Indigenous populations, especially those in remote areas without access to dental services. Though some programs emphasized Indigenous community involvement and ownership, their involvement was limited and varied among the programs. This suggests developing more shared space among Indigenous and non-Indigenous partners for strengthening integrated oral health care services. This might include participation in community activities and ceremonies such as sweat lodges, more involvement of elders and spiritual people, or more adoption of culturally sensitive healing practices.

Some included program descriptions did not describe performance evaluation, and this information gap constrains the comprehensive understanding of the extent of integration and its impact. Hence, there is a need for a comprehensive longitudinal evaluation of integrated primary oral health care intervention. Among selected program evaluations, a variety of outcome measures have been used. This points out the need for developing validated uniform measurement tools to evaluate integrated health care system performance. Furthermore, our results also identified the lack of validated indicators for measuring holistic oral health, in comparison to indicators available for biomedical oral health. This paucity warrants the development of indicators for better assessment of holistic oral health.

This review primarily identified integrated primary oral health care programs in North America and Australia. Nevertheless, there is a need to implement and conduct a subsequent evaluation of similar programs for Indigenous populations in other parts of the world, such as Asia, Africa, Europe, and South America.

## Conclusion

Study results suggest that implementing programs on the integration of oral health into primary health care has the potential to improve oral health-related outcomes for Indigenous populations. The array of approaches to integrated primary oral health care identified from the two-eyed seeing concept is relevant for Indigenous communities, with a particular emphasis on cultural integration. Most of the programs considered the variable degree of integration; however, more comprehensive integrated oral health care programs incorporating the holistic concept of health and oral health care are needed, to realize full effectiveness in Indigenous populations.

## Supplementary information

**Additional file 1.** Medline Search Strategy.

**Additional file 2.** Preferred Reporting Items for Systematic Reviews and Meta-Analyses Extension for Scoping Reviews (PRISMA-ScR) Checklist.

## Data Availability

All data generated or analyzed during this study are included in this published article (and its additional information files).

## Abbreviations

PCC: Population-Concept-Context
PRISMA-ScR: Preferred Reporting Items for Systematic Reviews and Meta-Analyses Extension for Scoping Reviews
IHWs: Indigenous Health Workers
COHI: Children’s Oral Health Initiative
