# Supplementary material for: Two-eyed seeing of the integration of oral health in primary health care in Indigenous populations: a scoping review
Source: Int J Equity Health. 2020 Jun 30;19:107. doi: 10.1186/s12939-020-01195-3 (PMC7329486; doi:10.1186/s12939-020-01195-3)
Supplement: Supplementary file 1 — Additional file 1. Medline Search Strategy. [file 12939_2020_1195_MOESM1_ESM.docx]

**Additional file 1.**

**Medline search strategy**

| # | **Searches** |
| --- | --- |
| 1 | (indigenous or aboriginal or "first nations" or metis or inuit*).mp. |
| 2 | ('Alaska* native' or 'native Alaska' or 'native American' or Mohawk or ojibw* or cree or dene or maori or 'torres strait islander*').mp. |
| 3 | Athapaskan.mp. or exp Indians, North American/ or exp Inuits/ or exp Health Services, Indigenous/ or exp Ethnopharmacology/ or Saulteaux.mp. or Wakashan.mp. or Cree.mp. or Aboriginal*.mp. or Indigenous*.mp. or Metis.mp. or off-reserve.mp. or onreserve.mp. or First Nation.mp. or First Nations.mp. or Amerindian.mp. or (urban adj3 (Indian* or Native* or Aboriginal*)).mp. or ethnomedicine.mp. or country food*.mp. or residential school*.mp. or (exp Medicine, Traditional/ not Chinese.mp.) or exp Shamanism/ or shaman*.mp. or traditional medicine*.mp. or traditional heal*.mp. or traditional food*.mp. or medicine man.mp. or medicine woman.mp. or autochtone*.mp. or (Native adj1 (man or men or women or woman or boy* or girl* or adolescent* or youth or youths or person* or adult or people* or Indian* or Nation or tribe* or tribal or band or bands)).mp. |
| 4 | 1 or 2 or 3 |
| 5 | exp Dental Health Services/ |
| 6 | Oral Health/ |
| 7 | Dentistry/ |
| 8 | Oral Medicine/ |
| 9 | exp Preventive Dentistry/ |
| 10 | exp Dental Facilities/ |
| 11 | exp Diagnosis, Oral/ |
| 12 | Stomatognathic Diseases/ |
| 13 | exp Mouth Diseases/ |
| 14 | exp Tooth Diseases/ |
| 15 | Pediatric Dentistry/ |
| 16 | exp Dentists/ |
| 17 | Community Dentistry/ |
| 18 | (dentist* or stomatology or Dental Prophylaxis or Fluoridation or Oral Hygiene or Oral Health or Dental Facilities or Dental Clinic* or Dental Office* or Oral Diagnos* or Mouth Disease* or Tooth Disease* or Dental Disease* or Dental Health Service* or Dental Service* or pedodontics).mp. |
| 19 | 5 or 6 or 7 or 8 or 9 or 10 or 11 or 12 or 13 or 14 or 15 or 16 or 17 or 18 |
| 20 | exp Primary Health Care/ |
| 21 | Primary Care Nursing/ |
| 22 | Primary Nursing/ |
| 23 | Physicians, Primary Care/ |
| 24 | (Primary care or Primary health care or Primary healthcare or Primary Nursing).mp. |
| 25 | 20 or 21 or 22 or 23 or 24 |
| 26 | exp "Delivery of Health Care, Integrated"/ |
| 27 | exp Community Health Services/ |
| 28 | (community care or community health care or community healthcare).mp. |
| 29 | 26 or 27 or 28 |
| 30 | Community Integration/ |
| 31 | systems integration/ |
| 32 | (Integrat* or Interprofessional or multidisciplin* or interdisciplin* or cooperat* or collaborat* or coordination*).mp. |
| 33 | ((Cross or multi or inter) adj (profession* or Disciplin*)).mp. |
| 34 | 30 or 31 or 32 or 33 |
| 35 | 4 and 19 and 25 and 34 |
| 36 | Limit 35 to (English) |
| 37 | (4 and 19 and 29 and 34) not 35 |
| 38 | Limit 36 to (English) |

*Derived from Hermina et al. 2017 [35]
